# *Announcement*: National Food Safety Education Month — September 2017

**DOI:** 10.15585/mmwr.mm6635a4

**Published:** 2017-09-08

September is National Food Safety Education Month. One of CDC’s food safety objectives is to raise awareness about healthy practices to prevent food poisoning. Every year in the United States, an estimated one in six persons (48 million persons) become ill, and 3,000 die from eating contaminated food (*1*). Some persons are at higher risk for foodborne illnesses (food poisoning) or might experience more severe symptoms: children aged <5 years (*2*), adults aged ≥65 years (*3*), pregnant women, and those with immune systems compromised by medical conditions, such as cancer, diabetes, and human immunodeficiency virus infection, or by treatments such as chemotherapy.

This year, CDC is focusing on raising awareness about these groups at high risk for foodborne illnesses. Persons in these groups should not eat undercooked animal products (e.g., meat, poultry, eggs, or seafood) (*4*), raw or lightly cooked sprouts, or unpasteurized milk and juices. They should also avoid eating soft cheese (e.g., queso fresco) unless the product’s label indicates that it was made with pasteurized milk.

Information about CDC’s activities related to Food Safety Education Month can be found at https://www.cdc.gov/foodsafety/education-month.html. Information on preventing food poisoning can be found at https://www.cdc.gov/foodsafety/groups/consumers.html.
